# 3.4 million real-world learning management system logins reveal the majority of students experience social jet lag correlated with decreased performance

**DOI:** 10.1038/s41598-018-23044-8

**Published:** 2018-03-29

**Authors:** Benjamin L. Smarr, Aaron E. Schirmer

**Affiliations:** 1 University of California at Berkeley, Department of Psychology, California, USA; 2 Northeastern Illinois University, Department of Biology, Chicago, USA

## Abstract

Misalignments between endogenous circadian rhythms and the built environment (*i.e*., social jet lag, SJL) result in learning and attention deficits. Currently, there is no way to assess the impact of SJL on learning outcomes of large populations as a response to schedule choices, let alone to assess which individuals are most negatively impacted by these choices. We analyzed two years of learning management system login events for 14,894 Northeastern Illinois University (NEIU) students to investigate the capacity of such systems as tools for mapping the impact of SJL over large populations while maintaining the ability to generate insights about individuals. Personal daily activity profiles were validated against known biological timing effects, and revealed a majority of students experience more than 30 minutes of SJL on average, with greater amplitude correlating strongly with a significant decrease in academic performance, especially in people with later apparent chronotypes. Our findings demonstrate that online records can be used to map individual- and population-level SJL, allow deep mining for patterns across demographics, and could guide schedule choices in an effort to minimize SJL’s negative impact on learning outcomes.

## Introduction

The human circadian system coordinates our many internal clocks to create a stable alignment of endogenous daily (circadian) rhythms to the environmental 24 hour day. Individuals show different stable phase alignments to the 24 h day (chronotypes), so that some people stably wake up and/or stay up later than others. Chronotype appears to be largely determined by the genetic composition of an individual’s circadian clock^1–5^. An individual may be able to choose to change their sleep/meal/activity time due to day-to-day schedule impositions, but they may not be able to shift their internal clocks in the same way, due to its genetic basis. This limit in flexibility is presumed to underlie the many ailments associated with jet lag (*e.g*., see^6,7^).

Misalignment between an individual’s circadian phase and their environment due to social imposition is called social jet lag (SJL)^8^. SJL is becoming more common^4^, and has been tied to many increased disease risks (*e.g*., see^8–10^). Currently, Western school schedules tend to be optimized for earlier chronotypes (*e.g*., 8 am–4 pm for primary and secondary school). Late chronotype students are at greater risk for persistent SJL relative to their academic environment, and this appears to result in decreased academic performance in primary school (*e.g*., see^11–13^), high school (*e.g*., see^14–16^), college (*e.g*., see^17–19^), and medical/professional school (*e.g*., see^20–22^).

Creating personal profiles of sleep and circadian rhythms for students is expensive and time consuming. This causes two major barriers to improving the way school schedules interact with learning. First, it limits our ability to create large scale maps of how SJL manifests over real-world populations, and this in turn limits our ability to assess how educational policies aimed at improving learning outcomes are hampered by SJL. Second, without the ability to make personal profiles automatically, it is impossible to generate profiles for the entire student population, which would allow for identification of at-risk students and the implementation of targeted interventions. We hypothesized that both of these hurdles could be overcome by data-mining online records of user activity in university learning management system (LMS) servers. These data accumulate without extra effort required by the students and without extra cost being incurred by the researcher, making them ideal for long-term use over large populations. We took advantage of existing infrastructure at NEIU to test whether online LMS data might indeed allow individual student profiles to be created over sizable populations, and provide insight into how SJL, imposed by schedule choice, impacts academic achievement in diverse, real-world student populations.

## Results

### Logins appear to contain circadian information, validated by known biological interactions

**Figure 1:**
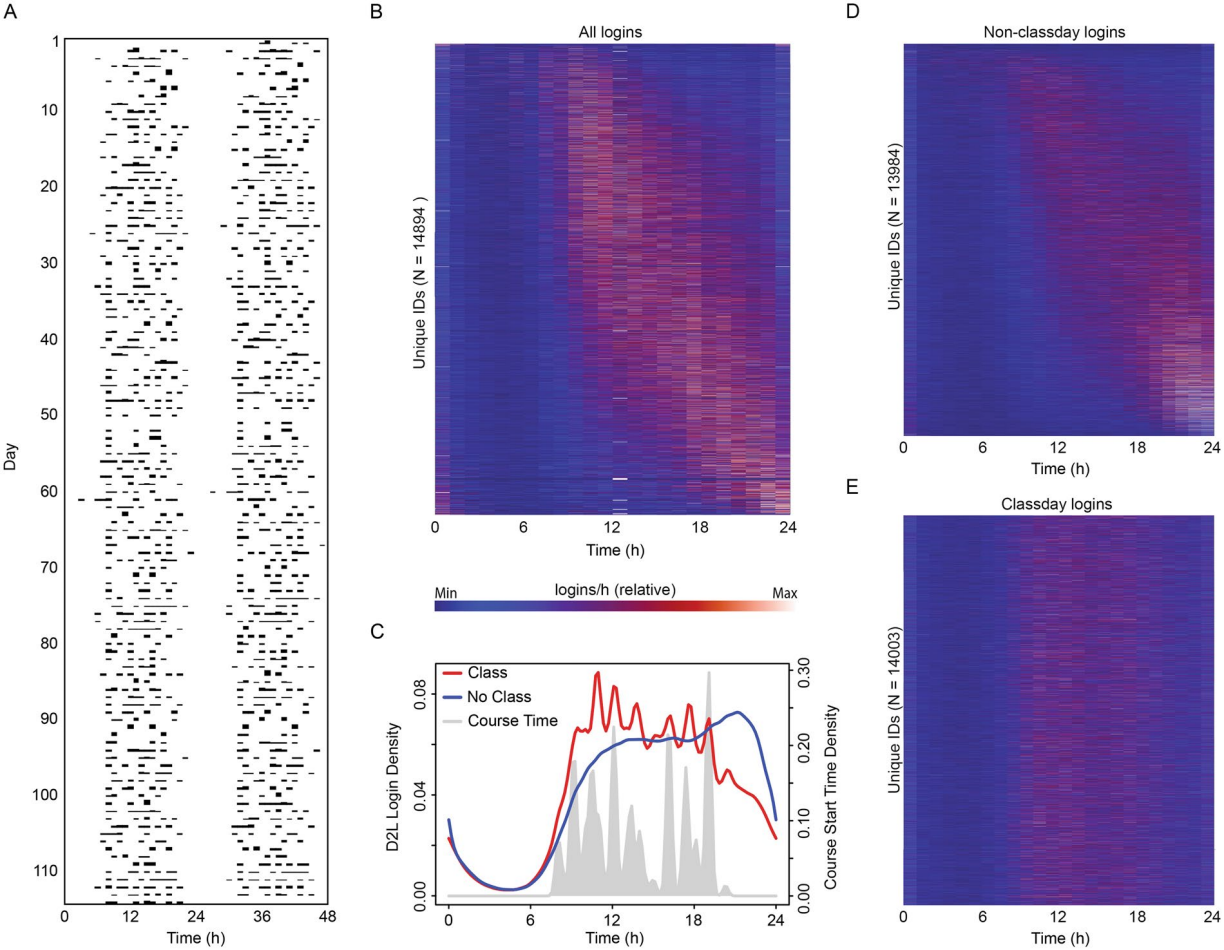
Student LMS logins showed signs that they contain circadian rhythm information, and social disruptions thereof. A double-plotted actogram (or raster plot) of login times from an example student across one semester (**A**) showed daily rhythms with an apparent sleep window between 23:00 and 06:00. Each student’s activity generated a unique hourly histogram (**B**, single row). When arranged by average phase of activity, a range of activity phases were apparent from early (**B**, top) to late (**B**, bottom), though all students shared an apparent sleep window in the late night and early morning. A histogram for all students on days with classes (**C**, red) showed crenulations that aligned to class start times (**C**, grey area). The same histogram for non-class days (**C**, blue) showed a delayed phase and no such crenulations. Consistent with the resulting supposition that non-class days are more representative of circadian rhythms, and class days more disruptive to circadian rhythms, histograms of activity on non-class days still showed a range of activity phases when sorted by average non-class day phase of activity (**D**), whereas the same ordering of individuals for class day activity (**E**) showed activity on class days to be largely during class times, and devoid of apparent chronotypes.

Data on LMS login activity from 14,894 unique students were analyzed for circadian-like patterns across 4 semesters, from fall 2014 to spring 2016 (excluding summer). There was a significant effect of time-of-day for logins across students (χ^2^ = 70862, *p* < 1 × 10^−308^). Daily patterns were clearly visible in individual double plotted actograms (Fig. 1A), which we transformed into daily histograms, by hour. Viewed together as a surface, with rows sorted by average phase-angle of activity (Fig. 1B), students exhibited a consistent probable-sleep window between 24:00 and 06:00–10:00, while the distribution of activity during active hours ranged from early to late, suggesting these data captured the expected range of chronotypes.

To explore these apparent chronotypes further, all student login events were separated within each individual into events on days for which that individual had a registered class, and events on days with no class. Because some classes did not have associated days in the digital records, the final number of students with logins on class days was 14,003, and the number of students with logins on non-class days was 13,984. Significant differences existed between the activity distributions on class and non-class days (Kolmogorov-Smirnov test, *D* = 0.11239, *p* < 2 × 10^−16^; Fig. 1C). On class days, the bulk of activity was in the first half of the day, with multiple peaks of activity (Fig. 1C, red line) corresponding closely to class times (Fig. 1C, grey peaks). By contrast, on non-class days the majority of activity was in the latter half of the day, lacked the class-associated crenulations, and displayed a delayed onset of activity (Fig. 1C, blue line). Given these differences, we took non-class day activity to be the more natural profile, and class days to be the SJL-imposing, constrained schedule. Consistent with this assessment, activity on non-class days showed a range of apparent chronotypes, with most activity later in the day and night (Fig. 1D), while by contrast, activity on class days, using the same order of individuals (Fig. 1E), showed no apparent chronotypes, and instead most activity was restricted to class times.

**Figure 2:**
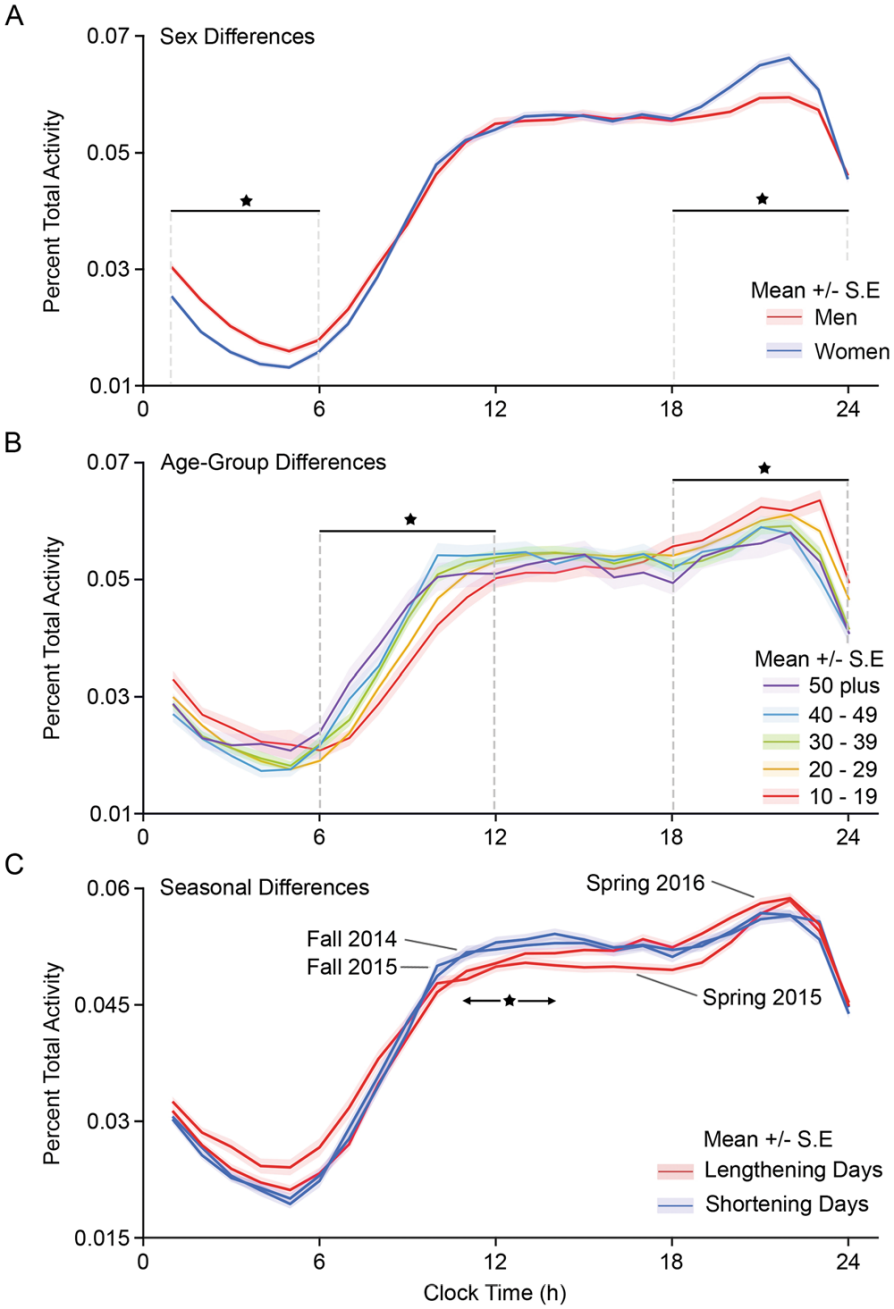
Non-class day activity distributions changed in ways expected of human circadian rhythms. Non-class day histograms sorted by gender (**A**) revealed that men (red) are more likely to stay active later than women (blue), with significantly increased activity between midnight and 06:00, while women showed increased activity in the evening (18:00–24:00). Sorted by decade of life (age, **B**), older students had significantly advanced phases, apparent both in earlier activity onset times (06:00–12:00), and decreased activity in the evening (18:00–24:00) for each additional decade of life. Sorted by season (**C**), there was a significant difference between fall semesters (blue) and spring semesters (red). Both fall semesters showed a consolidation of activity in the middle of the day, whereas both spring semesters showed a broader distribution of activity into the morning and evening. *p ≤ 0.05.

LMS logins are digital and not directly biological. To validate the hypothesis that login patterns on non-class days contain proxies for biological information, we assessed known interactions of phase with gender and age, and with expected interactions with seasonal change. Men have later sleep times than women^18,23,24^. Consistent with this, women (N = 5887) exhibited significantly more activity than men (N = 7890) in the evening (18:00–24:00, *F* = 143, *p* = 6 × 10^−33^), while men exhibited significantly increased activity relative to women at night (00:00–6:00, *F* = 47, *p* = 9 × 10^−12^, Fig. 2A).

Circadian phase also delays with puberty^25^, then advances with age^26–28^. Our cohort spans from teenagers to individuals in their 70s, with a median age of 25 yr (inner quartile range (IQR) = 9 yr). Comparison across decades of age (10–19, N = 2323; 20–29, N = 8770; 30–39, N = 2337; 40–49, N = 910; 50+, N = 509) revealed a significantly earlier phase of morning activity (06:00–12:00, *F* = 17.05, *p* = 5 × 10^−14^) and evening activity (18:00–24:00: *F* = 18.29, *p* = 5 × 10^−15^; Fig. 2B) for each additional decade of life.

Data reflecting seasonal changes to activity patterns in modern humans are sparse^29^. Because the data spanned two years, we assessed fall semesters (fall 2014, N = 8558; fall 2015, N = 8795) and spring semesters (spring 2015, N = 8795; spring 2016, N = 8051) for signs of recurrent seasonal change across years, on the assumption that humans would still show some seasonal pattern. Consistent with this expectation, in both years, the fall and spring showed significant differences (*F* = 10.6, *p* = 0.001; Fig. 2C) in login activity distribution. In the fall semesters, students exhibited increased activity in the middle of the day, consistent with the consolidation of activity under a shortening light phase. Conversely, in the spring semesters, student activity increased in the early and late day, consistent with the spreading of activity as the light phase lengthens. In summary, non-class days are validated as containing biological circadian information because they show expected changes with gender, age, and season.

### Social jet lag is strongly correlated with academic performance

**Figure 3:**
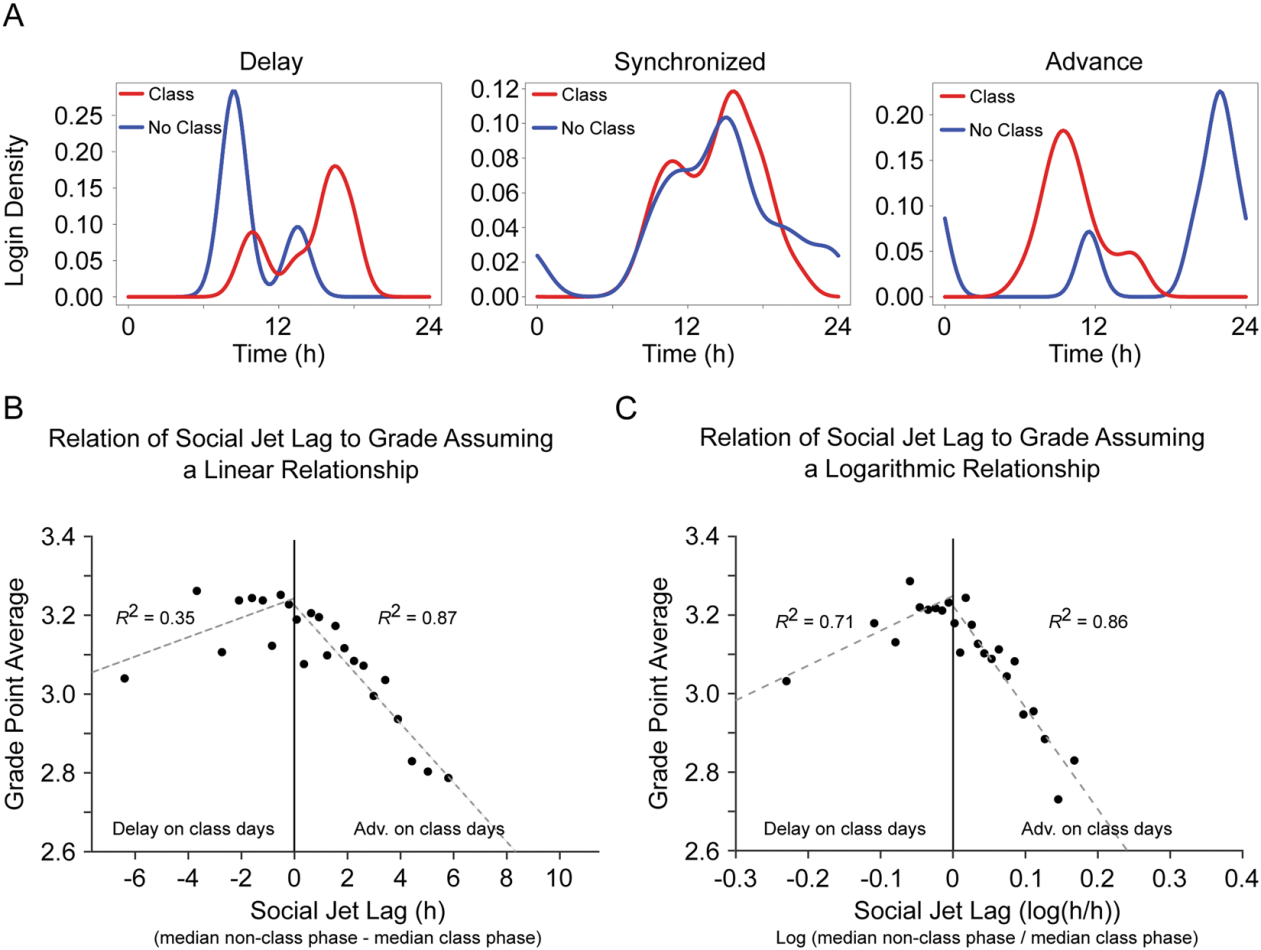
Social jet lag correlated with decreased academic performance for both advances and delays. Some students delayed from average non-class days (blue) to average class days (red) (**A**, left), some changed phase less than half an hour, on average, between non-class and class days (**A**, center), and some students advanced from average non-class days to average class days (**A**, right) (hourly histograms from example individuals shown for each condition). If SJL is calculated by linear subtraction (**B**), then amplitude of SJL showed a significant negative correlation with GPA for students who advanced on class days and a non-significant trend of correlation was apparent for students who delayed on class days. If SJL is calculated on a log scale (**C**), then amplitude of SJL showed a significant negative correlation with GPA for students who advanced on class days, and for students who delayed on class days (for both **B** and **C**, 24 groups are used, so that if SJL were random, 1 group would appear per hour of potential SJL).

SJL manifests in a difference of activity distribution as a function of day type (*e.g*., class or non-class days; see^18,30,31^). Given that we validated non-class days as containing circadian information, we predicted that greater SJL between class and non-class days would correlate with decreased academic performance, represented by lower grade point averages (GPAs). Overall, class days tend to have advanced phases when compared to non-class days (Fig. 1C). However, there is heterogeneity in the class:non-class day phase relationships of individuals. We found students with delayed, synchronized, and advanced class day phases (*i.e*., median login time was later, similar to, or earlier on class vs. non-class days, respectively; Fig. 3A). A comparison of phase within individuals revealed that only 40.4% of students are synchronized within one half hour of their class day phase, while 49.2% of individuals advanced, and 10.4% delayed their phases on class days by at least a half hour.

We quantified SJL as the difference between the average phase of activity on class days and non-class days. SJL is typically calculated as a linear difference (subtraction of phases; Fig. 3B). However, because biological systems often show non-linear, logistic rates of change^32–34^, we also calculated SJL by taking the log of the ratio of phases (Fig. 3C). We grouped all students into 24ths of the resulting SJL scale, under the assumption that if the data were random, then we would, on average, have one point per hour of possible shift. In both cases, the sign of regressions changed at SJL = 0, and so the correlation was calculated separately for positive and negative values of SJL. Both models found that phase advance on class days significantly correlated with decreased performance (linear: *R*^2^ = 0.87, *p* = 5 × 10^−7^; nonlinear: *R*^2^ = 0.86, *p* = 7 × 10^−7^). The nonlinear model also revealed a significant negative correlation of greater delays on class days with decreased performance (*R*^2^ = 0.71, *p* = 4 × 10^−3^), while the linear model found a trend (*R*^2^ = 0.35, *p* = 0.09).

### Social jet lag interacts with chronotype to shape academic performance

**Figure 4:**
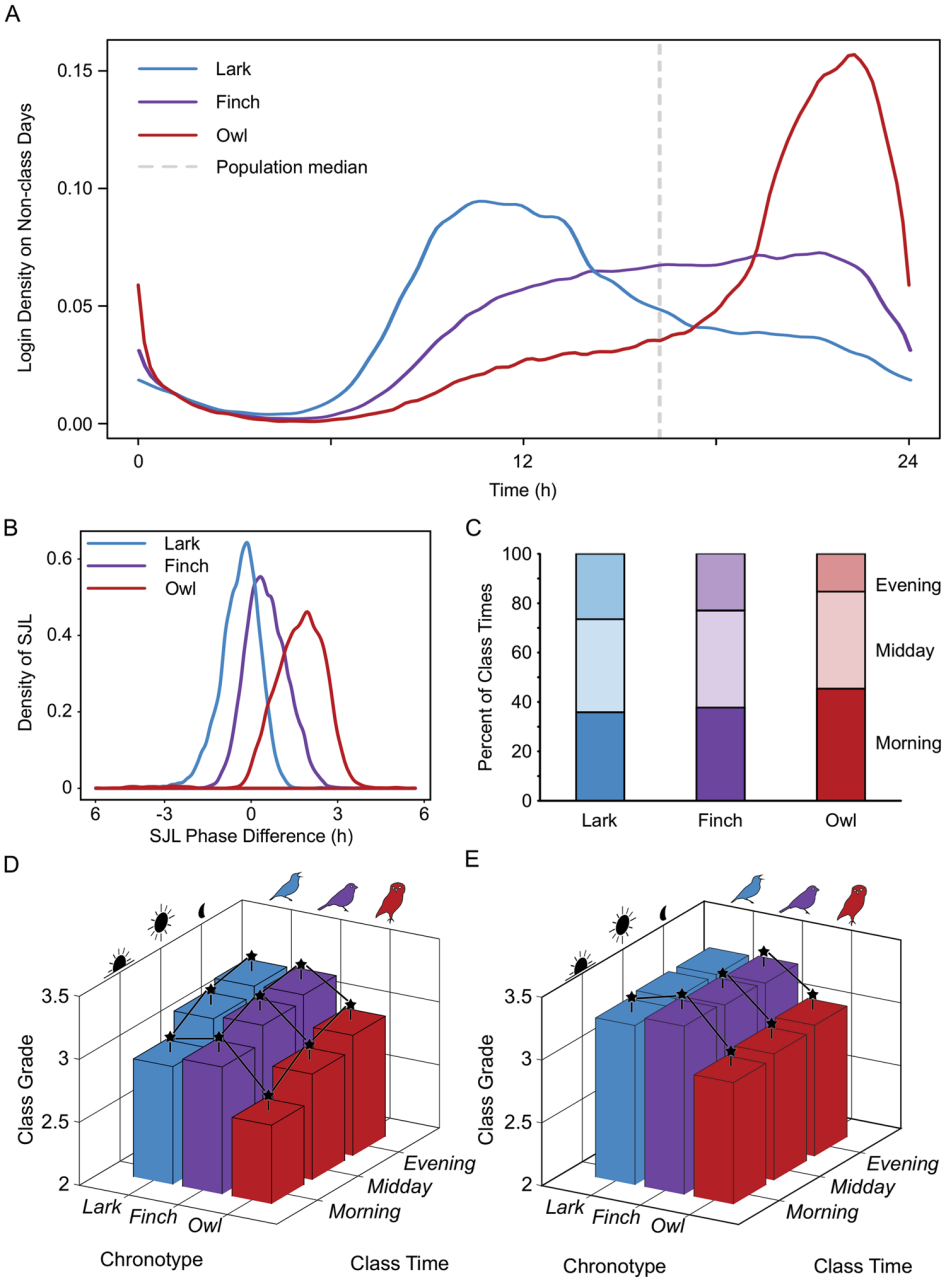
Owls had an academic disadvantage regardless of class start times. Average distribution of activity across non-class days for larks (blue), finches (purple), and owls (red) (**A**) revealed significant differences across the three chronotype categories. Owls experienced greater average SJL between non-class days and class days (**B**, red, linear SJL) than either larks (blue) or finches (purple). Owls also took a significantly higher proportion of morning classes than larks or finches (**C**) did. Analysis of class grades as a function of both chronotype category and class start time (**D**) revealed a significant disadvantage for owls across the day and a significant increase in class grade across the day for all chronotype categories. When time of day was normalized across all chronotype categories (**E**), then there is no longer an effect of time of day, and owls showed a significant disadvantage compared to larks and finches at all times of day. In plots D and E, the lowest bar represents the average of all morning class grades taken by individuals classified as owls. *p ≤ 0.05.

An early chronotype student taking only early morning classes and a late chronotype student taking only evening classes might both have an SJL score of 0. However, class schedules are not evenly distributed across the day, as morning classes were found to be more common (Fig. 1C,E). We hypothesized that, overall, early chronotypes would have an advantage, but that later chronotypes would have a relative advantage in later classes, when their SJL is lowest^35^. To test this, we assigned chronotypes to individuals based on whether their average non-class phase of activity was greater than 1 s.d. in advance of the population median (“larks”, N = 3857, median time = 12:46), within 1 s.d. of the population median (“finches”, N = 11261, median time = 16:22), or delayed more than 1 s.d. from the population median (“owls”, N = 3419, median time = 20:20). Daily profiles of the resulting chronotypes are significantly different (χ^2^ = 244860, *p* < 2.2 × 10^−16^; Fig. 4A). Owls and finches have a median advance on class days (1.74 h, IQR = 1.180 h; 0.49 h, IQR = 0.995 h, respectively). Larks have a median delay (0.32 h, IQR = 0.870 h; Fig. 4B).

We also categorized class start times as early (before 12:00), midday (12:00 to 17:00), or evening (after 17:00). Inauspiciously, owls took the highest percentage of morning classes (χ^2^ = 13.82, *p* = 0.001; Fig. 4C). All chronotypes exhibited significant academic improvement across the day (Fig. 4D; effect of time: F = 273, *p* = 1 × 10^−118^; larks: χ^2^ = 153, *p* = 7 × 10^−34^; finches: χ^2^ = 483, *p* = 1 × 10^−105^; owls: χ^2^ = 150, *p* = 2 × 10^−33^), with the average evening class grade points being at least 0.27 points higher than the average morning class grade points. Larks and finches significantly outperformed owls across the day (effect of chronotype: F = 391, *p* = 6 × 10^−170^). In the morning, all chronotypes differ (χ^2^ = 439, *p* = 6 × 10^−96^). By midday, only owls received significantly lower grade points (χ^2^ = 265, *p* = 2 × 10^−58^), and this effect continued in the evening (χ^2^ = 114, *p* = 2 × 10^−25^). In the morning, finches showed a small but significant advantage over larks (*p* < 0.05), consistent with our previous finding of decreased grades in students delaying themselves on class days.

The universal improvement in academic performance with later classes could arise for non-biological reasons, as in self-selection of different student groups or faculty cohorts for later classes. To control for this possibility, the comparisons were re-run on data with all grades normalized for each time of day, blinded to chronotype (Fig. 4E). Following this normalization, time of day no longer had a significant effect (F = 1.46, *p* = 0.2), refuting our hypothesis that owls would improve over the day while larks would worsen. Effect of chronotype remained significant, with similar overall differences as described above: owls showed a significant academic performance deficit at all class times (χ^2^ = 114, *p* = 2 × 10^−25^), and larks significantly underperformed finches only in the morning (*p* < 0.05). Available ACT scores were also compared between larks (N = 1227), finches (N = 4070), and owls (N = 1714) as a measure of educational development and college readiness (data not shown). No significant differences were found between the three groups (*p* = 0.06).

## Discussion

In this report, we took advantage of preexisting digital activity logs from 14,894 students across a 2-year period to map interactions of learning outcomes with SJL across a large, diverse population. Our analyses revealed that 60% of students experienced an average daily SJL of more than 30 min, with increasing amplitude correlating strongly with decreased academic performance, especially in late chronotypes. Those who delayed on class days display a less severe grade discounting than those who advanced on class days. This is consistent with phase advances being harder to adjust to than phase delays^36–38^, and with larks being more phase-aligned to daily school schedules than owls^39^, to the detriment of owls. Unfortunately, we saw no evidence that late chronotypes did proportionately better in their later classes.

If one assumes that healthy owls should do as well as their non-owl peers when performing at an optimal (later) phase, then our findings beg the question: what factors impede owls from reaching this level of parity? We suggest that persistent instability (from a student’s schedule) causes an increase in internal desynchrony^40–42^. Internal desynchrony, briefly, is a loss of stable phase relationships, as multiple internal oscillators move through time at different periods; in the context of jet lag, this allows eventual realignment of phases. We suspect that this cannot be overcome day-to-day, making academic achievement at any phase more difficult while this desynchrony persists. If this is true, a more stable schedule should lead to improved performance even in owls. At NEIU, over 50% of classes meet only two days per week, with start times ranging from 08:00 to 20:30, contributing to schedule instability. In contrast, most high school start (and end) times are consistent day to day, and in high school, late chronotypes have been shown to improve in later classes^35^. If the owl disadvantage was exacerbated by schedule instability, then the owls we analyzed should not show the same degree of disadvantage in their high school performance. Consistent with this hypothesis, we found no significant differences when comparing available ACT scores from high school. Further research is needed to quantify the contributions of chronotype and SJL frequency to academic performance across different stages of education, and to confirm or refute this proposed root of the observed owl disadvantage.

One of the advantages of a data-mining approach like the one used here is that it enables exploration of phenomena that can only be seen when data have high temporal resolution, and also cover a long period of recording. To this point, we uncovered a novel pattern of stably-repeating seasonal changes in how people distribute their activity across the day, hour by hour. This finding suggests that many other phenomenological questions in chronobiology might be informed by a similar data mining approach, using other preexisting data sets. A further advantage of mining pre-existing data from academic institutions is that such data are generated independent of any study, without recourse to questionnaires or personal logs/diaries or wearable sensors, and without the associated limitations (cost, labor, compliance, *etc*.,) and biases (recall, inclusion, self-selection, *etc*.). Of course, the disadvantage is that these data are not directly measuring biological outputs like sleep or circadian melatonin level, but rather represent time spent specifically on academically-targeted effort of some sort. We believe these data are validated as proxies for rhythmic biological information by reflecting characteristic changes in daily rhythms by gender and age (and novel but apparently naturalistic seasonal changes). Exactly what validates each novel form of data should remain an open discussion. Because we analyzed pre-existing data, it was not possible to run deeper validations on a smaller cohort of these students, but such an effort in the future would obviously be informative. Assuming these data are reflecting biological information, we feel their academic nature makes them qualitatively different from other data-mining efforts, such as analyses of data scrubbed from social media sources (*e.g*., see^43–45^**)**. For the purposes of assessing academic performance, we believe these data are more appropriate, but the relative merits of different data sources for more social or more academic applications requires further exploration.

At the time these data were generated, NEIU was ranked the most diverse university in the midwest^46^, and so our data appear to confirm the importance of circadian stability and phase in academic performance across a wide range of ages and demographic variables. It therefore seems reasonable to advocate that circadian rhythms and SJL should be considered in school and class scheduling by institutions as well as by individuals. Universities might want to provide chrono-counseling to students about the importance of chronotype and daily stability to academic achievement, but it is difficult to know who to target or how to give advice, in large part due to the difficulty in gathering information about individual students’ daily patterns of sleep and activity. The data source type used here (LMS) generates data for universities in-house, and so similar data would be available for building within-campus chrono-counseling capabilities, without the issues of privacy and security that might arise from seeking personal data from outside sources (as in, scrubbing social media feeds). However, these data do not contain high enough login frequencies to reliably generate actograms that capture an individual’s phase every single day. Therefore, coupling this sort of analysis with outside data sources or wearable sensors would allow increased resolution and predictive power in future work. At the same time, data-mining approaches like the ones reported here could be carried out over very large populations (there are roughly 20 million college and university students in the US**)**^47^, allowing for deeper mining and more sensitive pattern detection. Either at large scales, or with more detailed personal data, such work has the potential to enable data-based, personal counseling within the education sphere similar to efforts being used to generate personal, predictive medicine in the clinical sphere^48,49^. There are many non-trivial problems between the current state of knowledge and the realization of this goal, but the benefits to individuals and societies stemming from enhancing education by enabling individuals to take advantage of their own biological rhythms are surely substantial.

## Materials and Methods

### Data Collection and Deidentification

All experiments were performed in accordance with relevant guidelines and regulations under the Northeastern Illinois University institutional review board (NEIU IRB) approved protocol #16-073. Pre-existing login time data for 4 semesters (excluding summer) from fall 2014 to spring 2016 were collected from the university LMS Desire2Learn® (https://www.d2l.com/). At NEIU, the LMS system is used for many activities including but are not limited to: Viewing course materials, grades, and assignment feedback; completing and submitting assignments online; participating in online discussion boards; and contacting the instructor or classmates. LMS login data was combined with student data from the student information system Banner® (http://www.ellucian.com/student-information-system/). Student data included both demographic (gender and age) and academic (semester GPAs, courses taken, start and end times of individual courses, individual course grades, and ACT® scores) variables. The Office of Institutional Research and Assessment (IRA) at Northeastern Illinois University collected both sets of data and used the student Banner IDs to merge the files. IRA then deidentified the data by striping the student banner IDs and replaced them with pseudo ID numbers based on a novel algorithm that was maintained at IRA and not shared with the authors. Because these data were preexisting and deidentified, they meet the qualifications for exemption as defined by 45 CFR 46.101(b) and informed consent was not obtained. Raw data files are available upon request and with the approval of the NEIU IRB.

### Data Processing and Graphing

The raw data received from IRA were initially processed in the R statistical package^50^, and subsequent analyses were carried out with both R and Matlab. The data were separated by semester and all date and time values were converted from text using the “stringr” R package (http://stringr.tidyverse.org) into the formats of M/D/Y and H:M:S, respectively. Time values were used to derive circular time-as-radians measures for each LMS login event, with a circular period of 24 hours. Each date was compared against the individual student’s class schedule as well as the Northeastern Illinois University’s Academic Calendar (http://www.neiu.edu/academics/academic-catalog) and each day (and corresponding login events) were designated as occurring on a “class day” or “non-class day.” Semester GPAs were calculated by converting individual letter grades into their numerical equivalent grade points (A = 4.0), which were then averaged for each student each semester. Median radial login phase was calculated for class days and non-class days for each individual each semester using the circular statistics toolbox (http://bethgelab.org/software/circstat/) for Matlab. Demographic information within the records was used to sort individuals’ histograms by gender and age. If an individual was missing a demographic descriptor (*e.g*., no age listed), then that individual was excluded from the analysis of that demographic. Averages of histograms grouped by demographic variables were calculated using means, as medians generated discontinuous outcomes that were not representative of daily distributions.

Median phases were used to define each individual’s chronotype, and to determine their amount of social jet lag. All individuals within each semester were classified as a lark, finch, or owl. Larks were defined as those with median non-class day phases from more than one standard deviation below the group median to pi radians from that median. Owls were defined as those with median non-class phases from more than one standard deviation above the group median to pi radians later than that median. Individuals within one standard deviation of the group median were designated as finches. For individuals that were represented across multiple semesters (4286 students were represented in all 4 semesters, 2185 where only in 3, 4533 were only in 2, and 3890 were only in 1), chronotype and corresponding analyses were independently generated for each semester. Social jet lag was determined in two ways, and both are reported in the results section. The first assumed a linear relationship, subtracting the non-class day median phase from the class day median phase for each individual, yielding a number of hours of median phase-shift from non-class days to class days. The second approach assumed a nonlinear relationship, using the log of the ratio of the median phase on non-class days to the median phase on class days.

### Statistics

All statistical analyses were conducted in the R statistical package or Matlab. Data were assumed to be non-normal, and non-parametric Kruskal-Wallis tests were used where possible for group comparisons (indicated by χ^2^ score). For radial statistics, as in median phase, clock time (24 h) is converted into 2 pi radians, so that angular characteristics can be taken across all login events; this radial approach avoids artifacts such as the average of 23:59 and 00:01 being 12:00 noon. The Kolmogorov-Smirnov test was used for non-parametric comparisons of the shape of continuous distributions (named when used). Multiple-way comparisons were needed to test for the effect of chronotype and class time on class grades. As Kruskal-Wallis tests cannot easily be converted to N-way comparison tests, N-way ANOVAs were used (indicated by F scores). To approximate normality for these tests, data were converted to z-scores either across all groups (as one group, for raw comparison, see Fig. 4D) or within class times (as three groups, with all chronotypes represented in each, see Fig. 4E), to control for time-of-day effects on grade (see Results: SJL interacts with chronotype to shape academic performance). Post-hoc comparisons were Kruskal-Wallis for individual group comparisons, and Dunn’s tests for individual time-point comparisons (threshold set to *p* < 0.05). In all cases, test statistics were Bonferroni-corrected based on the number of comparisons. All regressions used a linear model.
